# Quantification and Classification of Diclofenac Sodium Content in Dispersed Commercially Available Tablets by Attenuated Total Reflection Infrared Spectroscopy and Multivariate Data Analysis

**DOI:** 10.3390/ph14050440

**Published:** 2021-05-07

**Authors:** Eirini Siozou, Vasilios Sakkas, Nikolaos Kourkoumelis

**Affiliations:** 1 Laboratory of Analytical Chemistry, Department of Chemistry, University of Ioannina, 45 110 Ioannina, Greece; irene.siozou7@gmail.com; 2 Department of Medical Physics, School of Health Sciences, University of Ioannina, 45 110 Ioannina, Greece; nkourkou@uoi.gr

**Keywords:** diclofenac sodium, ATR spectroscopy, Fourier transform infrared, quantitative analysis, chemometrics

## Abstract

A new methodology, based on Fourier transform infrared spectroscopy equipped with an attenuated total reflectance accessory (ATR FT-IR), was developed for the determination of diclofenac sodium (DS) in dispersed commercially available tablets using chemometric tools such as partial least squares (PLS) coupled with discriminant analysis (PLS-DA). The results of PLS-DA depicted a perfect classification of the tablets into three different groups based on their DS concentrations, while the developed model with PLS had a sufficiently low root mean square error (RMSE) for the prediction of the samples’ concentration (~5%) and therefore can be practically used for any tablet with an unknown concentration of DS. Comparison with ultraviolet/visible (UV/Vis) spectrophotometry as the reference method revealed no significant difference between the two methods. The proposed methodology exhibited satisfactory results in terms of both accuracy and precision while being rapid, simple and of low cost.

## 1. Introduction

**Figure 1:**
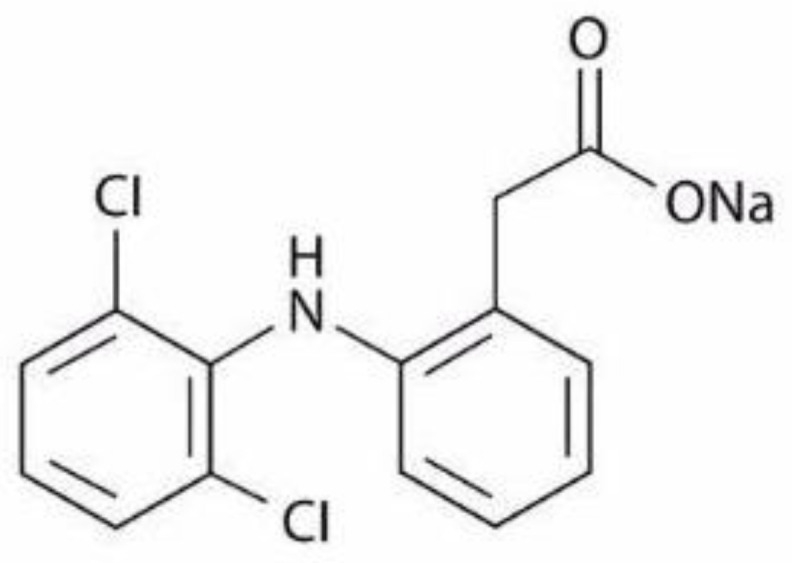
Structure of diclofenac sodium molecule.

Non-steroidal anti-inflammatory drugs (NSAIDs) are a broad family of compounds which are commonly used for the treatment of inflammation and pain. Diclofenac ([2-(2,6-dichloroanilino)phenyl]acetic acid) is one of the most widely used NSAIDs commonly used for the treatment of rheumatoid arthritis, osteoarthritis, ankylosing spondylitis, and musculoskeletal and sports injuries, as well as for the relief of dysmenorrhea and postoperative pain [1,2,3,4,5,6]. It is typically found as sodium or potassium salt and it is available in a variety of drug formulations including tablets, capsules, ophthalmic drops, suppositories, and ointments, depending on the site of application. Diclofenac sodium (DS) (Figure 1) is primarily used for its anti-inflammatory properties and acts at a slower pace than potassium salt which is readily absorbed by the body and provides instant relief [7].

Quality control of dosage forms plays a major role in pharmaceutical industries, since the products must meet certain criteria set by pharmacopoeias and other authorities; otherwise, they may be substandard and/or even pose a threat to public health. Therefore, the development of sensitive and reliable methods for the determination of active ingredients in dosage forms is crucial. The official method for the determination of DS proposed by the European Pharmacopoeia (Ph.Eur.) and the United States Pharmacopoeia (USP) includes the dispersion of the standard compound in anhydrous acetic acid followed by titration with perchloric acid and potentiometric determination of the endpoint [8,9], while for the determination of DS in slow-release tablets, the USP proposes its dispersion in a methanol–water mixture (70:30) and subsequent analysis in a liquid chromatograph with a UV detector, using methanol and phosphate buffer (pH 2.5) as mobile phase components [10]. In recent decades, several analytical methods have been developed for the determination of diclofenac in drug products, including spectrophotometric [7,11,12,13,14,15,16], spectroscopic [17,18,19,20,21], fluorimetric [22], potentiometric [22,23,24,25,26,27], and chromatographic methods [28,29,30,31,32,33,34].

Spectroscopic methods have the advantages of a relatively low cost and rapid sample analysis. Attenuated total reflectance infrared spectroscopy with Fourier transformation (ATR FT-IR) is not considered as a quantitative technique by many. However, ATR FT-IR has been used before as a quantitative technique for the analysis of NSAIDs as an accurate, sensitive, and low-cost technique. 

Van Overbeke et al. used ATR FT-IR to quantify ketoprofen in pharmaceutical formulations such as capsules and injection ampoules, in combination with partial least-squares (PLS) analysis [35]. Similarly, Boyer et al. proposed an analytical method without prior sample treatment for the determination of niflumic acid in a pharmaceutical gel by ATR FT-IR and PLS calibration with a root mean square error of prediction (RMSEP) equal to 0.2 for the validation set [36]. Lawson et al. examined the potential use of ATR FT-IR to provide rapid quantitative analyses of suspect counterfeit tablet formulations containing paracetamol [37]. Sensitive methods based on ATR FT-IR spectroscopy were developed by Hassib et al. for the quantitative assessment of four NSAIDs, namely, etodolac, tolfenamic acid, bumadizone, and diacerein, by peak profiling using normal or derivative spectroscopy [38].

PLS combined with ATR FT-IR was proposed by Silva et al. for the simultaneous determination of the antibiotics sulphamethoxazole and trimethoprim in raw material powder mixtures [39]. Kandhro et al. developed a reliable analytical method for the quantitative assessment of cefixime in orally administered pharmaceutical formulations based on ATR FT-IR spectroscopy. Standards were prepared in aqueous medium ranging from 350 to 6000 mg/kg and PLS regression was applied in the range 1485–887 cm^−1^ [40]. Furthermore, ATR FT-IR has also been used for the determination of polymorphs in dosage forms. Salari and Young investigated the feasibility of ATR FT-IR for the qualitative and quantitative analysis of mixtures of three known polymorphs of ganciclovir, an antiviral compound. Quantitation was carried out using PLS and the results proved the method to be sufficient for the intended purpose [41]. Helmy et al. applied ATR FT-IR for the identification and quantitation of two polymorphs of aprepitant, a substance P antagonist for chemotherapy-induced nausea and vomiting, in solid pharmaceutical tablets. A detection limit of 3 wt % of one polymorph over the presence of another was reported in these compact binary samples [42]. According to these results, the combination of chemometric tools with ATR FT-IR shows great promise as a quantitative technique to boost quality assurance in the pharmaceutical industry and elsewhere.

The quantification of DS in tablets using the technique of ATR FT-IR was first reported by Mazurek et al., who performed PLS modeling based on ATR FT-IR as well as FT-Raman spectra of the commercial products containing 25–100 mg of DS [20]. In that work, it was suggested that the quality of the constructed models depended on the homogeneity of the active pharmaceutical ingredient (API) distribution, noting that the ATR FT-IR technique was more susceptible to quantification errors. The same authors also investigated the quantitative determination of DS in other dosage forms using FT-Raman spectroscopy [17,18], and diffuse reflectance IR spectroscopy (DRIFTS) [21].

In this work, we report the use of PLS and PLS-discriminant analysis (PLS-DA) for the detection and quantification of DS by ATR FT-IR spectroscopy. The optimized method was applied as a proof of concept in commercially available tablets and exhibited excellent results in terms of both accuracy and precision. We argue that ATR FT-IR spectroscopy can achieve high prognostic efficacy when methanolic solutions are used combined with multivariate analysis applied on a concisely selected wavelength subset of the mid-infrared region.

## 2. Results and Discussion

### 2.1. Development of the Chemometrics Model

**Figure 2:**
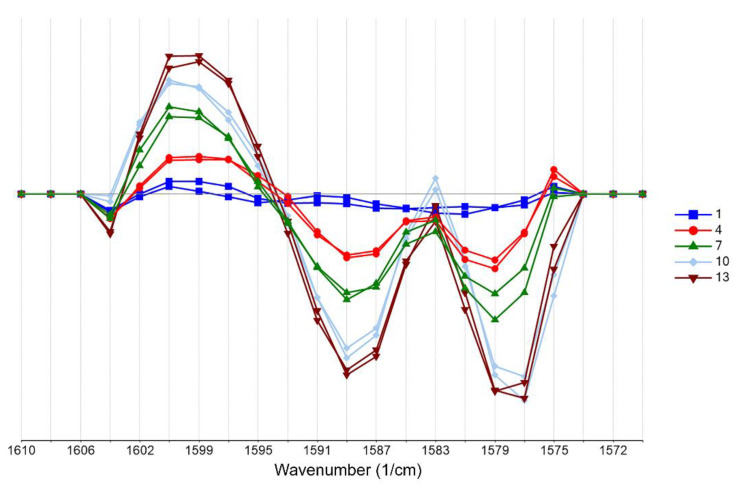
Second derivative transformation with Savitzky–Golay smoothing focusing on the 1600–1500 cm^−1^ region of the spectra of the training set (blue: 1 g/L, red: 4 g/L, green: 7 g/L, cyan: 10 g/L, brown: 13 g/L of DS).

**Figure 3:**
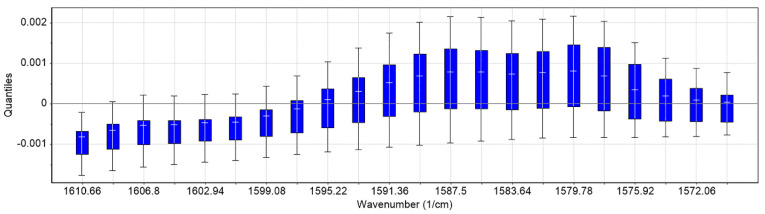
Boxplots (quantiles) of the training set. The maximum intensity deviation is observed in the region 1595–1575 cm^−1^.

Second derivative differentiation coupled with seven smoothing points (Savitzky–Golay) was applied to the spectra of the training set to enhance resolution and eliminate possible linear baseline drift between samples (Figure 2). Second derivative spectroscopy has the advantage of producing sharp vibration bands while offering resolution enhancement of overlapping peaks. However, as differentiation increases, the signal noise is typically combined with a Savitzky–Golay filter. The spectra analysis focused on the region ~1600–1500 cm^−1^, which is assigned to the –COO- group. As can be seen in Figure 2 and Figure 3, differences owing to the DS concentration changes of the training set (standard solutions, 1–13 g/L) are apparent in this region (region of interest).

Multivariate analysis of the spectra was employed to quantitatively determine the DS content. Our approach was driven by the characteristic carboxylate (COO-) vibration band of the DS molecule. Specifically, the asymmetric stretching vibration is assigned to the 1600–1500 cm^−1^ region [43]. In this specific area, a gradual increase in absorbance intensity followed the increased DS concentration in both standard and tablet solutions (Figure 2 and Figure S1). However, a number of excipients that could potentially absorb in the region of interest, especially molecules with the carboxylic ion, still exist in the tablet. These could, in theory, interfere with our analysis. We found that such possible excipients in our case are magnesium stearate and stearic acid. Previous work [44,45] showed that both of them do not absorb in our selected region of interest (1600–1575 cm^−1^). Moreover, they exist in typically low concentrations (~5–6% compared to the API) in the various tablet formulations and therefore are either below the limit of detection or their contribution is cancelled out as a “matrix” residual error in the analysis. Finally, in this work, we used second derivative spectroscopy for resolution enhancement of possible overlapping spectral bands. We did not determine additional bands that could have obscured our data. 

**Figure 4:**
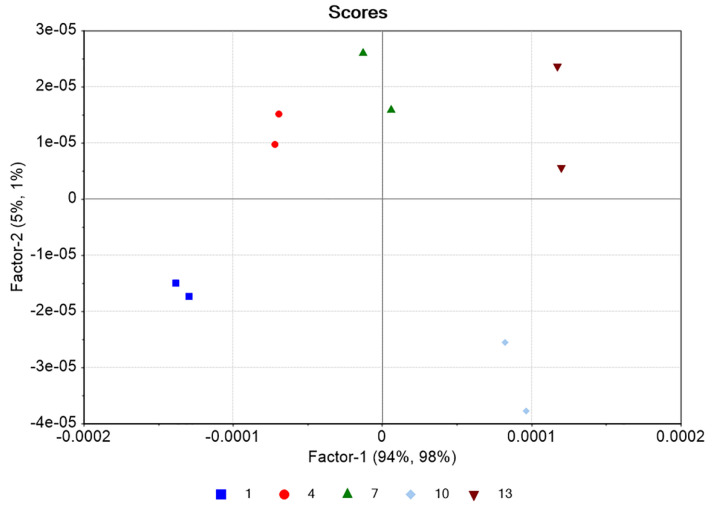
Scores plot of the training set (blue: 1 g/L, red: 4 g/L, green: 7 g/L, cyan: 10 g/L, brown: 13 g/L of DS). Each point corresponds to a different control batch consisting of three spectra for each concentration (N_total_ = 30 spectra).

**Figure 5:**
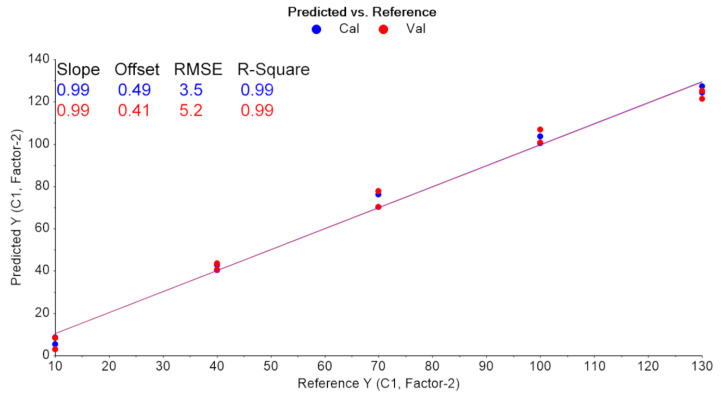
Leave-one-out CV results (blue: training set, red: validation set).

**Figure 6:**
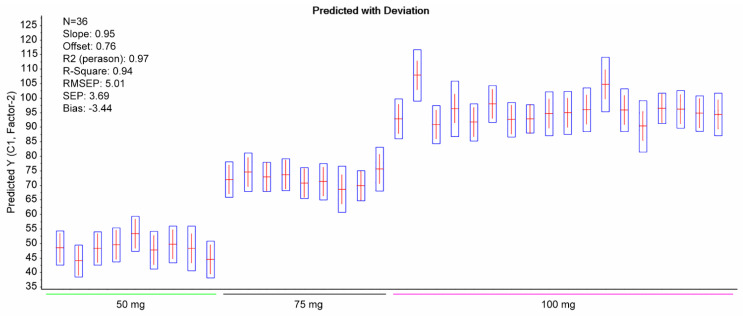
Predicted content of DS in the test set alongside deviation (boxplots). The statistical outcomes of the PLS model are also shown.

PLS regression analysis with leave-one-out cross-validation resulted in the scores plot depicted in Figure 4, where only two factors explain most of the variance. Using two-factors analysis, the leave-one-out cross-validation procedure yielded a correlation coefficient equal to R^2^ = 0.99, with a root mean square error (RMSE) of approximately 5% (Figure 5). The histogram of the residuals (Figure S2 of the Supplementary Material) shows that the data are not skewed. Therefore, the constructed prediction model correctly classifies the training set samples for the cross-validation, while the standard error of calibration (SEC) reveals minimum bias. The developed PLS model was applied to the test set spectra (N = 36), and the results are shown in Figure 6, where the predicted values are depicted with deviation. The numerical predicted concentrations are given in Table 1 and Table S1 of the Supplementary Material.

The effects of the analysis among the three different classes of formulations (50, 75, and 100 mg) were all significant (*p* < 0.001) at the 95% confidence level, according to one-way Welch’s or Brown–Forsythe’s ANOVA (Table S2 of the Supplementary Material).

For the classification, the precision and recall had the value of 1, yielding a flawless classification. The PLS model revealed RMSEP = 5.01 and R^2^ = 0.94 for the test set. 

### 2.2. Comparison with Reference Method

The results obtained by the proposed method were compared to those obtained by the reference method (UV spectrophotometry) (Table 2, Tables S3 and S4 of the Supplementary Material). Welch’s *t*-test was applied in order to find out if the results are statistically significantly different. The results are shown in Table 3, in which it is noted that t_exp_ < t_crit_ (for a two-tailed test); therefore, the null hypothesis stating that there is no significant difference between the results of the two methods is valid.

## 3. Materials and Methods

### 3.1. Samples and Reagents

Three types of commercial tablets (two original formulations of 50 and 75 mg (standard and sustained release, respectively), one generic of 100 mg (sustained release)) were purchased from local pharmacies and stored at room temperature in the dark until their analysis. All tests were performed within product expiry dates. Standard diclofenac sodium (DS) of 100.0% purity was purchased from Calbiochem. The solvents, methanol for residual analysis and LC-MS-grade water, were purchased from Carlo Erba reagents and Merck, respectively.

### 3.2. Preparation of Training Set

For the development of the chemometric model, the IR spectra of DS solutions with known content were used to calculate a calibration model. Thus, we performed a pilot trial to investigate which vibrational peaks are most sensitive to the quantification of DS. A stock standard solution of DS was prepared by dissolving 0.16 g of pure substance in 10 mL of methanol (16 g/L). From this solution, calibration standards containing 1–13 g/L of DS were prepared by dilution with methanol. The training set contained N = 33 reference spectra from two different batches of calibration standards. 

### 3.3. Preparation of the Test Set

Multiple tablets of each batch (18 from 50 and 75 mg and 18 from 100 mg formulations) were weighed on an analytical balance and the average weight was determined. The tablets were finely ground in a mortar and quantities of approximately the average tablet weight were transferred into 10 mL volumetric flasks which were completed to volume with methanol. The resulting solutions were sonicated for 10 min, centrifuged for 15 min, and filtered using a 0.45 μm syringe filter (Whatman™). The filtrates, i.e., the test set, were stored in 8 mL glass vials.

### 3.4. ATR FT-IR Analysis

**Figure 7:**
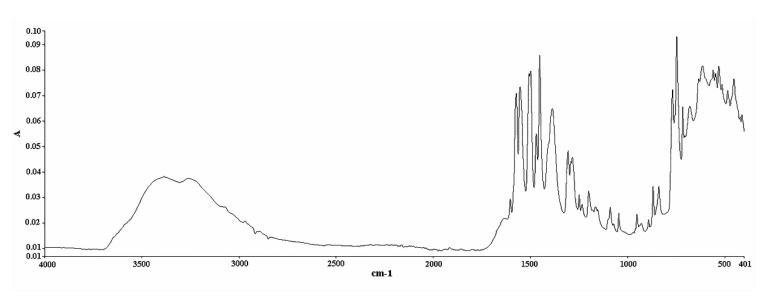
ATR FT-IR spectrum of DS (standard compound) in powder form (abscissa units: wavenumber (cm^−1^); ordinate units: absorbance).

**Figure 8:**
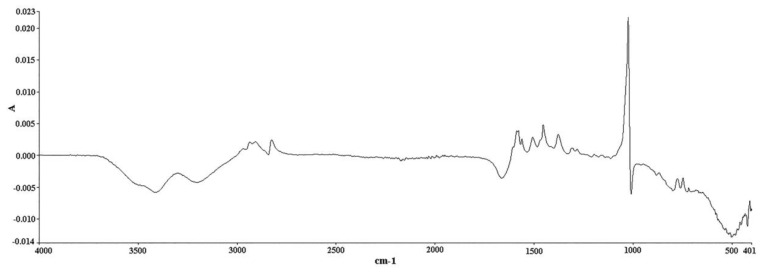
ATR FT-IR spectrum of DS methanolic solution (20 g/L) after taking a methanol spectrum as background spectrum (abscissa units: wavenumber (cm^−1^); ordinate units: absorbance).

The analysis was conducted on a Spectrum Two FT-IR apparatus equipped with a diamond ATR accessory (Perkin Elmer, Waltham, MA, USA). IR spectra were recorded in absorbance units. The spectral zone analyzed was between 4000 and 400 cm^−1^ with a resolution of 4 cm^−1^ and a data interval of 1.93 cm^−1^. It is worth mentioning that the time needed for the completion of one single measurement under these conditions was only 76 s. An amount of 100 μL of methanol (solvent) was placed on the diamond crystal and scanned as the background spectrum. Subtracting the spectrum of the pure solvent from the sample spectrum is a typical procedure to eliminate the solvent contribution. However, this contribution is minimal in our case, i.e., in the 1600–1500 cm^−1^ region (Figure S3 of the Supplementary Material), it has no negative effect on the followed methodology, and it allows the use of multiple solvents to effectively dissolve the tablets, provided that their spectra do not significantly interfere with the region of interest. The same quantity of the prepared samples was placed on the crystal afterwards and scanned in the same conditions. The measurement of each solution was repeated in triplicate, and the response mean value was calculated (Figure 7 and Figure 8). Multivariate analysis was performed using the Unscrambler X 10.3 software (CAMO Analytics SA).

### 3.5. Multivariate Analysis

PLS-DA was used for the classification of the tablets into three different groups based on their DS concentrations as an initial crude rapid discrimination test. Then, a PLS model was constructed to predict the samples’ concentration. PLS-DA is a PLS regression of a set of binary variables on a set of predictor variables. We used three classes for the discrimination of samples into the 50, 75, and 100 mg DS concentrations. PLS-DA searches the components or latent variables that discriminate among different groups of samples’ spectra (X matrix) according to their maximum covariance with the Y-variables, as the target class (Y matrix). Y was specified as a one-dimensional number to represent different DS concentrations in the model. The algorithm PLS2 was adopted in this work as the model classified the samples in three classes of DS concentration. The outcomes were evaluated by computing precision and recall as follows: (tp/(tp+fp)), and (tp/(tp+fn)) (true positives (tp), false positives (fp), false negatives (fn)), respectively. A PLS regression model was constructed and validated using leave-one-out cross-validation to determine the optimal matrix rank for the regression. The predictive ability of the PLS model was assessed by the root mean square error of prediction (RMSEP) and the coefficient of determination (R^2^). 

### 3.6. Reference UV/Vis Analysis

UV/Vis spectrophotometry was employed as the reference method, by measuring the absorbance of DS at λ_max_ = 280 nm, as described by Fabre et al. (1993) [46]. A stock standard solution of DS (60 mg/L) was prepared by dissolving 3 mg of standard in 50 mL of solvent mixture (methanol–water, 1:1 *v/v*). Serial dilutions were performed for the preparation of calibration standards containing 5–15 mg/L DS. These solutions were measured in the range 200–500 nm and the calibration curve was constructed by using their absorbance at λ_max_. The measurements were conducted using a UV-1800 spectrometer (Shimadzu, Kyoto, Japan). The known samples that were prepared with the aforementioned procedure (§ 3.3) were further diluted with the solvent mixture, in order for their final concentrations to fall within the calibration curve concentration range.

## 4. Conclusions

In the present study, the feasibility of a new analytical methodology based on ATR FT-IR spectroscopy for the quantitative determination of DS in commercially available tablets was investigated. The novelty of the work is that it utilizes only a small portion of the spectral range. Specifically, it focuses on the COO- vibration which manifests within less than 100 wavenumbers, in the region of ~1600–1500 cm^−1^. In contrast to published studies that applied multivariate analysis to the whole spectrum, we focused on a small region which typically has a superior signal-to-noise ratio, does not suffer from overlapping peaks, and does not require extensive pre-processing which is user-specific and therefore error-prone. Moreover, the small region of interest can significantly speed up the chemometrics calculations in an industrial setting with vast datasets and can possibly be used independently for all pharmaceutical formulations that contain the carboxylate ion in the API. The results show that the classification of tablets regarding their API content was consistent, while PLS regression analysis led to the construction of an effective chemometric model that can be readily used for the quantification of DS in practically any tablet. Furthermore, the results of the proposed method were compared to those obtained by a reference method, and the *t*-test showed that there is no significant difference between the results of the two methods, proving the suitability of the new method for the intended purpose.

## Figures and Tables

**Table 1:** The mean DS content of the tablets (N = 36) as specified in the patient’s leaflet and the corresponding calculated values of the same tablets’ DS content based on the PLS model.

DS Content	Calculated (Mean ± SD)
50 mg	48.10 ± 2.12
75 mg	72.03 ± 1.73
100 mg	95.55 ± 2.20

**Table 2:** The mean DS content values of the commercial tablets calculated by the reference UV/Vis method.

DS Content	Calculated (Mean ± SD) UV/Vis
50 mg	49.31 ± 0.48
75 mg	74.29 ± 0.64
100 mg	98.79 ± 1.06

**Table 3:** Welch’s *t*-test.

	Paired *t*-Test	
	Variable 1 (ATR FT-IR)	Variable 2 (UV)
Mean	71.89	74.13
Deviation	562.89	612.17
Sample size	3	3
Pearson correlation	0.99	
Hypothetical mean difference	0	
Degrees of freedom	2	
*t*	3.81	
P(T ≤ *t*) one-tailed	0.03	
*t* critical, one-tailed	2.92	
P(T ≤ *t*) two-tailed	0.06	
*t* critical, two-tailed	4.30	

## Data Availability

The data presented in this study are available on request from the corresponding author.

## Supplementary Materials

The following are available online at https://www.mdpi.com/article/10.3390/ph14050440/s1, Figure S1: The 1600–1500 cm−^1^ band of the standard solutions, depicting the increase in absorbance as a result of the increase in concentration, Figure S2: Residuals plot of the test set. As the residuals are gathered near zero and have a random pattern, we conclude that the data are fitted in an accurate model, even when the two outliers (in the case of 100 mg tablets) are included, Figure S3: FTIR spectrum of pure methanol as measured (blue) and with beaseline subtraction. A minor contribution of the solvent exists in the 1600–1500 cm^−1^ region. Table S1: Predicted concentrations of the known samples’ solutions (test set), Table S2: ANOVA significance test and data summary, Table S3: UV calibration curve data.
