# Supplementary material for: Quantification and Classification of Diclofenac Sodium Content in Dispersed Commercially Available Tablets by Attenuated Total Reflection Infrared Spectroscopy and Multivariate Data Analysis
Source: Pharmaceuticals (Basel). 2021 May 7;14(5):440. doi: 10.3390/ph14050440 (PMC8151404; doi:10.3390/ph14050440)
Supplement: Supplementary file 1 [file pharmaceuticals-14-00440-s001.zip › pharmaceuticals-1165893-supplementary.pdf]

Quantification and classification of diclofenac sodium content in dispersed commercially available tablets by Attenuated Total Reflection Infrared Spectroscopy and Multivariate Data Analysis

Eirini C. Siozou[a], Vasilios A. Sakkas [a] and Nikolaos Kourkoumelis [b]*

[a]Laboratory of Analytical Chemistry, Department of Chemistry, University of Ioannina, 45 110 Ioannina, Greece

[b]Department of Medical Physics, School of Health Sciences, University of Ioannina, 45 110 Ioannina, Greece

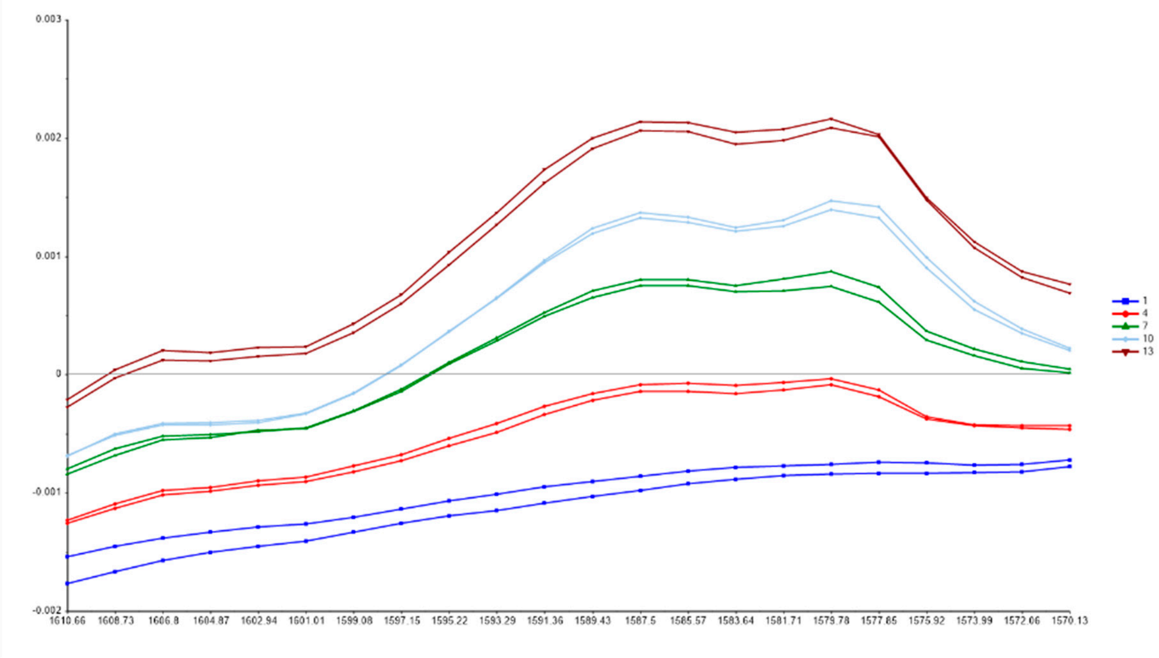

Figure S1. The 1600-1500 cm-1 band of the standard solutions, depicting the increase in absorbance as a result of the increase in concentration.

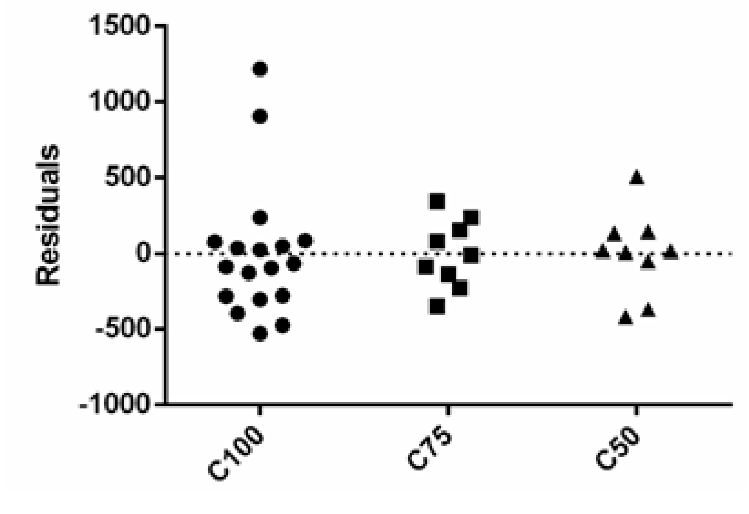

Figure S2: Residuals plot of the test set. As the residuals are gathered near zero and have a random pattern, we conclude that the data are fitted in an accurate model, even when the two outliers (in the case of 100 mg tablets) are included.

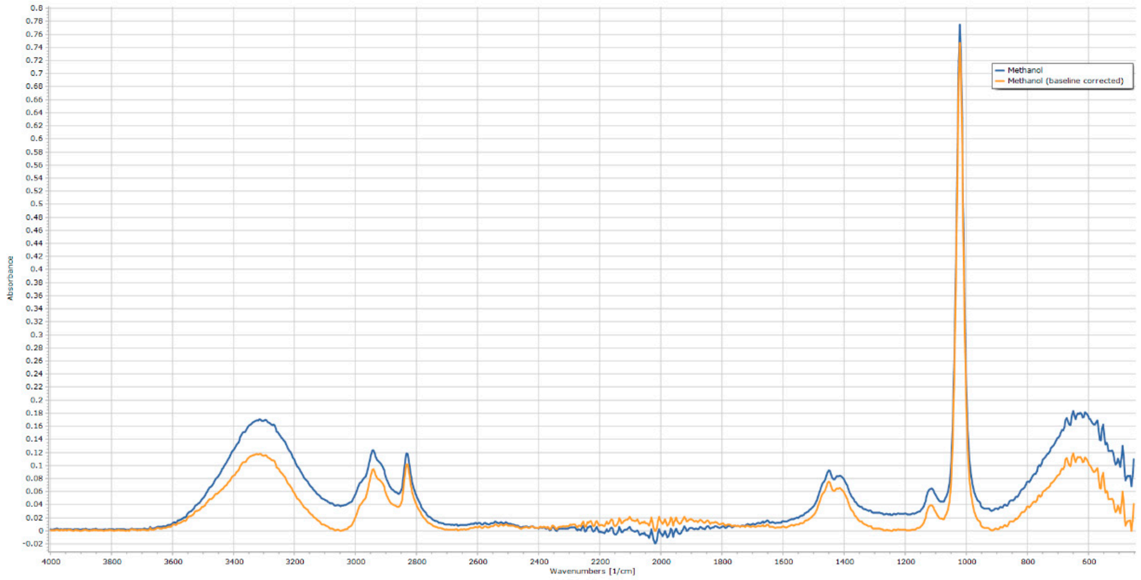

Figure S3. FTIR spectrum of pure methanol as measured (blue) and with beaseline subtraction. A minor contribution of the solvent exists in the 1600–1500 cm^{-1} region.

Table S1: Predicted concentrations of the known samples' solutions (test set).

	REAL SAMPLES	PREDICTED CONCENTRATIONS OF THE SOLUTIONS (mg/L)	DEVIATION	PREDICTED TABLET CONTENT (mg)
**Tablets 50 mg**	SOLUTION #1-1	4831.688	583.7999	48.3 ± 5.8
	SOLUTION #1-2	4391.424	547.1805	43.9 ± 5.5
	SOLUTION #1-3	4826.427	571.7602	48.3 ± 5.7
	SOLUTION #2-1	4943.451	587.332	49.4 ± 5.9
	SOLUTION #2-2	5319.096	603.9611	53.2 ± 6.0
	SOLUTION #2-3	4760.168	651.2797	47.6 ± 6.5
	SOLUTION #3-1	4956.626	637.6409	49.6 ± 6.4
	SOLUTION #3-2	4816.241	768.8667	48.2 ± 7.7
	SOLUTION #3-3	4442.204	635.6401	44.4 ± 6.4
**Tablets 75 mg**	SOLUTION #1-1	7190.304	606.1545	71.9 ± 6.1
	SOLUTION #1-2	7439.536	661.3398	74.4 ± 6.6
	SOLUTION #1-3	7282.211	496.72	72.8 ± 5.0
	SOLUTION #2-1	7357.887	548.5912	73.6 ± 5.5
	SOLUTION #2-2	7067.717	536.5697	70.7 ± 5.4
	SOLUTION #2-3	7116.051	624.7159	71.2 ± 6.2
	SOLUTION #3-1	6853.65	792.2275	68.5 ± 7.9
	SOLUTION #3-2	6975.644	517.4031	69.8 ± 5.2
	SOLUTION #3-3	7547.586	752.9271	75.5 ± 7.5
**Tablets 100 mg**	SOLUTION #1-1	9276.764	683.5461	92.8 ± 6.8
	SOLUTION #1-2	10774.98	890.885	107.8 ± 8.9
	SOLUTION #1-3	9080.44	657.1057	90.8 ± 6.6
	SOLUTION #2-1	9630.198	952.634	96.3 ± 9.5
	SOLUTION #2-2	9160.615	641.7767	91.6 ± 6.4
	SOLUTION #2-3	9792.374	635.0111	97.9 ± 6.4
	SOLUTION #3-1	9250.944	593.6955	92.5 ± 5.9
	SOLUTION #3-2	9273.151	488.0576	92.7 ± 4.9
	SOLUTION #3-3	9459.782	755.0127	94.6 ± 7.6
	SOLUTION #4-1	9489.07	742.6256	94.9 ± 7.4
	SOLUTION #4-2	9590.018	751.2822	95.9 ± 7.5
	SOLUTION #4-3	10461.48	940.5557	104.6 ± 9.4
	SOLUTION #5-1	9577.55	738.7823	95.8 ± 7.4
	SOLUTION #5-2	9026.071	885.2714	90.3 ± 8.9
	SOLUTION #5-3	9639.369	531.3099	96.4 ± 5.3
	SOLUTION #6-1	9603.998	648.1289	96.0 ± 6.5
	SOLUTION #6-2	9468.948	609.9111	94.7 ± 6.1

SOLUTION #6-3	9426.261	731.3464	94.3 ± 7.3

Table S2: ANOVA significance test and data summary

Brown-Forsythe ANOVA test			
F* (DFn, DFd)		719.5 (2.000, 32.32)	
P value		<0.0001	
P value summary		****	
Welch's ANOVA test			
W (DFn, DFd)		566.7 (2.000, 20.29)	
P value		<0.0001	
P value summary		****	
Data summary			
Number of treatments (columns)		3	
Number of values (total)		36	
	Conc. of solution of 100 mg tablets (mg/L)	Conc. of solution of 75 mg tablets (mg/L)	Conc. of solution of 50 mg tablets (mg/L)
Number of values	18	9	9
Minimum	9026	6854	4391
25% Percentile	9268	7022	4601
Median	9479	7190	4826
75% Percentile	9632	7399	4950
Maximum	10775	7548	5319
Mean	9555	7203	4810
Std. Deviation	442.6	224.9	276.6
Std. Error of Mean	104.3	74.97	92.21
Lower 95% CI	9334	7031	4597
Upper 95% CI	9775	7376	5022

Table S3: UV calibration curve data.

λ_{max}	:	280 nm
Calibration curve equation	:	y = 0.0777x + 0.0282
Correlation coefficient	:	R^2 = 0.9993
Concentration range	:	5 – 15 ppm
S_a	:	0.001194
S_b	:	0.01261
$S_{y/x}$	:	0.00909

Table S4: Results of known samples by UV/Vis spectrophotometry.

REAL SAMPLE	ABSORBANCE	ESTIMATED VALUE (mg)	DECLARED VALUE (mg)
#50-1	0.798	49.54	50
#50-2	0.786	48.76	50
#50-3	0.799	49.63	50
#75-1	0.802	74.75	75
#75-2	0.790	73.56	75
#75-3	0.801	74.55	75
#100-1	0.797	98.90	100
#100-2	0.787	97.68	100
#100-3	0.804	99.80	100
